# Generation of a multipurpose *Prdm16* mouse allele by targeted gene trapping

**DOI:** 10.1242/dmm.029561

**Published:** 2017-07-01

**Authors:** Alexander Strassman, Frank Schnütgen, Qi Dai, Jennifer C. Jones, Angela C. Gomez, Lenore Pitstick, Nathan E. Holton, Russell Moskal, Erin R. Leslie, Harald von Melchner, David R. Beier, Bryan C. Bjork

**Affiliations:** 1 Department of Biochemistry, Chicago College of Osteopathic Medicine, Midwestern University, Downers Grove, IL 60515, USA; 2 Department for Molecular Hematology, University Hospital Frankfurt, Goethe University, 60590 Frankfurt am Main, Germany; 3 Department of Molecular Biosciences, The Wenner-Gren Institute, The University of Stockholm, SE-106 91 Stockholm, Sweden; 4 Department of Orthodontics, College of Dentistry, The University of Iowa, Iowa City, IA 52242, USA; 5 Department of Anatomy, Chicago College of Osteopathic Medicine, Midwestern University, Downers Grove, IL 60515, USA; 6 Department of Pediatrics, University of Washington School of Medicine, Seattle, WA 98105, USA; 7 Center for Developmental Biology and Regenerative Medicine, Seattle Children's Research Institute, Seattle, WA 98105, USA

**Keywords:** Conditional gene trap, Cleft palate, Mandible, Micrognathia, Pierre Robin sequence

## Abstract

Gene trap mutagenesis is a powerful tool to create loss-of-function mutations in mice and other model organisms. Modifications of traditional gene trap cassettes, including addition of conditional features in the form of Flip-excision (FlEx) arrays to enable directional gene trap cassette inversions by Cre and Flpe site-specific recombinases, greatly enhanced their experimental potential. By taking advantage of these conditional gene trap cassettes, we developed a generic strategy for generating conditional mutations and validated this strategy in mice carrying a multipurpose allele of the *Prdm16* transcription factor gene. We demonstrate that the gene trap insertion creates a null mutation replicating the Pierre Robin sequence-type cleft palate phenotype of other *Prdm16* mutant mice. Consecutive breeding to *Flpe* and *Emx1^IREScre^* deleter mice spatially restricted *Prdm16* loss to regions of the forebrain expressing the homeobox gene *Emx1*, demonstrating the utility of the technology for the analysis of tissue-specific gene functions.

## Abstract (precis)

**Summary:** Described is the first targeting of an invertible gene trap to generate a conditional *Prdm16* mouse allele and its use to assess phenotypic consequences of *Prdm16* loss during craniofacial and brain development.

## INTRODUCTION

To better understand human development and disease, the role of single genes must be examined in the context of the entire organism. Efforts to expand our understanding of molecular mechanisms involved in human development and disease require the systematic analysis of gene function in model organisms such as mice. The availability of the mouse genome sequence and strong conservation at nucleotide and amino acid levels make mice ideally suited for studying the phenotypic consequences of altered gene expression.

A variety of strategies have been employed to introduce specific alterations into the mouse genome. Of these, chemical mutagenesis using *N*-ethyl-*N­*-nitrosourea (ENU) and gene targeting or trapping in mouse embryonic stem (ES) cells has been used extensively in ‘forward’ and ‘reverse’ genetic screens, respectively (Stottmann and Beier, 2010). By exploiting site-specific recombinase systems such as Cre/*loxP* and Flpe/*Frt* in combination with gene targeting or trapping, it became possible to induce spatially and/or temporally controlled mutations in the mouse for functional studies (Branda and Dymecki, 2004; Lao et al., 2012).

Gene trapping is an unbiased, high-throughput approach for inducing loss-of-function mutations and is performed with gene trap vectors that simultaneously mutate and report the expression of the trapped gene at the site of insertion (Kitajima and Takeuchi, 1998; Stanford et al., 2001). Classic gene trap vectors comprise a strong 5′ splice acceptor (SA) site, a promoterless reporter and/or selectable marker gene and a 3′ polyadenylation site, which are traditionally introduced into mouse ES cells via electroporation or viral infection to randomly insert throughout the genome. If insertion occurs into an intron of an expressed gene in the correct reading frame, the endogenous transcript is terminated by the gene trap's polyA site, resulting in a fusion transcript from which the reporter is translated along with a truncated and non-functional version of the endogenous protein. Traditional gene traps induce null mutations and require a minimum level of endogenous gene expression for event selection (Friedel and Soriano, 2010; Friedrich and Soriano, 1991). To add conditional features to gene trap vectors, Schnütgen et al. (2005) equipped a series of gene trap vectors with flip-excision (FlEx) arrays that enable directional gene trap cassette inversions by Flpe and Cre recombinases (Schnütgen et al., 2005). Because these gene traps generate multipurpose alleles enabling a variety of post-insertional modifications, they have been used extensively in combination with gene targeting or trapping by the International Knockout Mouse Consortium (IKMC) in an effort to conditionally mutate every protein coding gene of the mouse genome (Bradley et al., 2012). To pursue a gene of interest by taking advantage of the gene trap features to simultaneously inactivate and report gene expression at the insertion site, Friedel et al. (2005) used homologous recombination to introduce gene trap cassettes into a pre-specified gene, a strategy referred to as ‘targeted trapping’.

Here, we describe a mouse transgenic strain carrying a *Prdm16* multipurpose allele created by the targeted insertion of the conditional gene trap cassette FlipROSAβgeo* into the second intron of *Prdm16*. PRDM16 is a transcription factor whose inactivation has severe developmental consequences. It was first identified as an oncogene activated in cases of myelodysplastic syndrome and acute myeloid leukemias (Mochizuki et al., 2000). Loss-of-function *Prdm16* mutations were shown to affect brown adipose cell fate, and hematopoietic and neuronal stem cell maintenance, and have been linked to the cardiomyopathy developing in patients with 1p36 deletion syndrome (Aguilo et al., 2011; Arndt et al., 2013; Chuikov et al., 2010; Seale et al., 2008, 2007). Our group and others have demonstrated a role for *Prdm16* during mandible and palate development. Its mutational inactivation in mice causes cleft secondary palate (CP) by a similar mechanism as seen in Pierre Robin sequence (PRS)-type clefting (Bjork et al., 2010; Warner et al., 2007). PRS is evident in humans exhibiting micrognathia and glossoptosis, which are palate-extrinsic factors that precipitate the development of cleft palate (Tan et al., 2013).

We demonstrate that the targeted insertion of the gene trap induces a null mutation that can be rescued and re-induced in pre-specified cells and tissues by consecutive matings to *Flpe* and *Cre* deleter mice. By simultaneously mutating and reporting gene expression at the insertion site, the strategy seems ideally suited for the analysis of tissue-specific gene functions at the organismal level.

## RESULTS

### Vector design and targeted trapping of *Prdm16* in ES cells

**Fig. 1.**
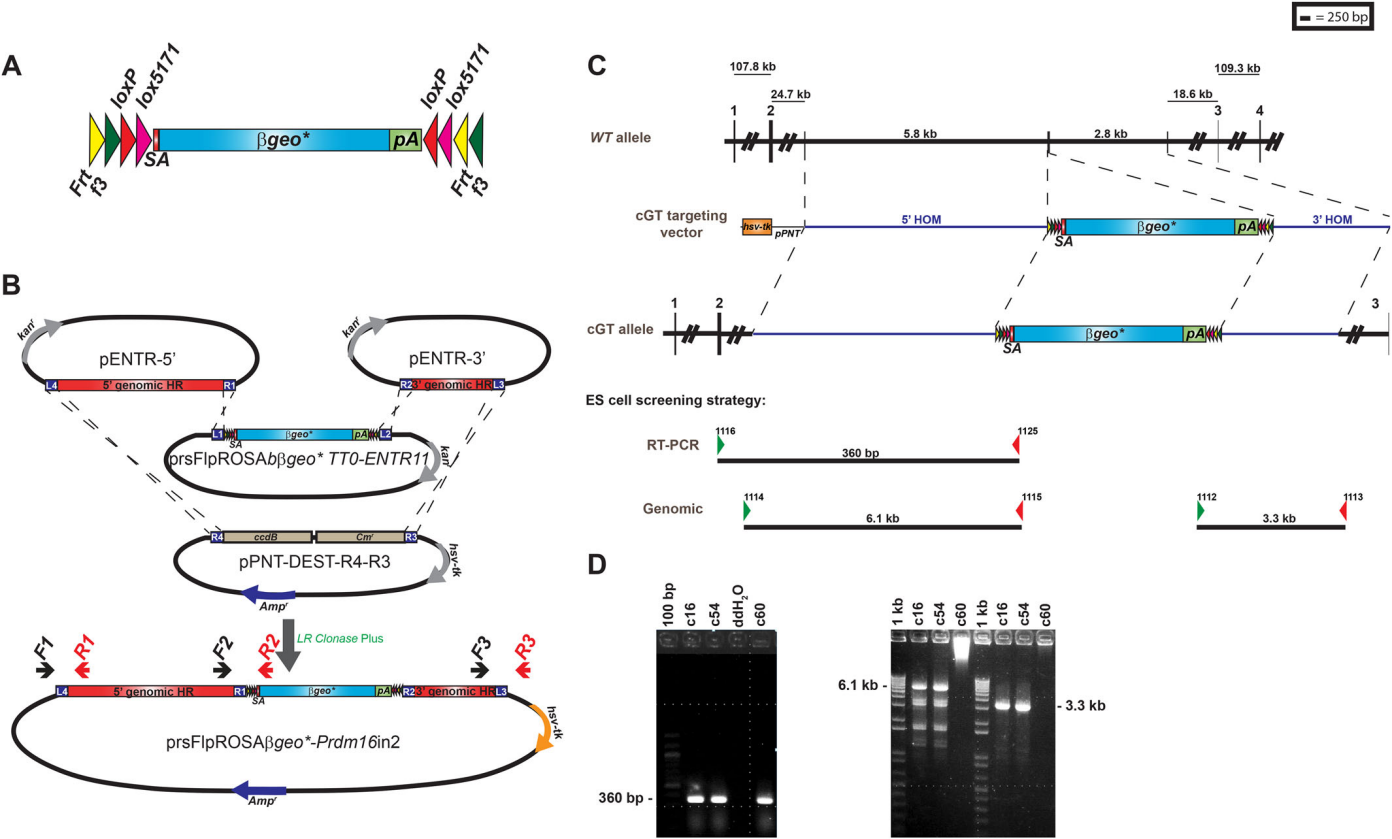
**Overview of cGT vector construction and generation of the targeted multifunctional *Prdm16^cGT^* null allele.** (A) Schematic representation of the multifunctional gene trap cassette adapted from Schnütgen et al. (2005). Colored arrowheads flanking the βgeo* fusion gene represent wild-type (wt) and mutant pairs of *Frt* (*Frt* and *f3*) and *loxP* (*loxP* and *lox5171*). (B) Schematic representation of the multi-fragment GATEWAY recombination strategy used to generate targeted trapping constructs to produce multifunctional gene trap alleles in the mouse. The method employs PCR amplification of 5′ and 3′ genomic homology regions (HR) using primers that contain appropriate ‘*att*’ sites for site-specific BP Recombinase reactions with GATEWAY Donor (DONR) plasmids to produce 5′ and 3′ HR Entry (ENTR) plasmids. These plasmids are combined with a third ENTR clone that contains the reading-frame-specific prsFlipROSAβgeo* multifunctional gene trap cassette and a modified pPNT gene targeting plasmid converted for use as a GATEWAY Destination (DEST) plasmid in *in vitro* LR Recombinase reactions. The result was the final targeted trapping plasmid containing the multifunctional gene trap cassette flanked by 5′ and 3′ HRs in the pPNT plasmid backbone. Red and black arrows depict the oligonucleotide primers used to amplify across the recombination junctions for screening of bacterial transformants for verification of the desired targeting construct (F1/R1, F2/R2 and F3/R3). (C) Homologous recombination of the multifunctional cGT gene trap cassette into the *Prdm16* locus within intron 2. Oligonucleotide primers used for screening G418-resistant ES clones by RT-PCR (to identify trapped *Prdm16* cGT fusion transcript) or genomic PCR [to confirm correct targeting at the 5′ and 3′ homology regions (HRs)] are depicted along with the expected product sizes. (D) Confirmation of ES clones c16 and c54 by RT-PCR from ES clone total RNA (left) and PCR amplification from ES clone genomic DNA (right). Clone c60 was positive via RT-PCR screening; however, the correct 5′ and 3′ genomic products were not detected. pA, polyadenylation; SA, splice acceptor.

Plasmid vectors were designed to facilitate the efficient generation of targeted trapping constructs comprising a FlipROSAβgeo* gene trap cassette and *Prdm16*-specific homology arms (Friedel et al., 2005; Komada et al., 2000; Schnütgen et al., 2005). A modified MultiSite GATEWAY cloning approach modeled after previous work described by Ikeya et al. (2005) was used (Fig. 1B). Three Entry clones [5′ and 3′ genomic homology arm clones and the FlipROSAβgeo* clone (Schnütgen et al., 2005)] as well as one Destination clone (a modified gene targeting plasmid) were constructed, each containing appropriate attachment (*att*) sites to allow *in vitro* site-specific recombination to produce the desired gene targeting construct. 5′ and 3′ homology arms of ∼5.8 kb and ∼2.8 kb, respectively, were PCR-amplified using oligonucleotide primers containing specific *att* sites at their 5′ ends using the MICER clone MHPN 168g07, which contains an ∼9 kb genomic DNA insert derived from the 129S5 mouse strain, as template DNA (Adams et al., 2004). These purified *att*-site-flanked PCR products were recombined with GATEWAY Donor clones (pDONR-P4-P1R and pDONR-P2R-P3) in *in vitro* Clonase reactions to generate 5′ and 3′ homology arm Entry clones (pENTR-5′HOM and pENTR-3′HOM). Highly conserved genomic sequences contained within each clone were sequenced to verify the integrity of the amplified DNA fragments and guard against the possibility of introducing functional sequence variants upon ES cell targeting.

The FlipROSA*βgeo* gene trap cassettes (prsFlipROSA*βgeo*) were modified to obtain three plasmid variants allowing reading frame continuity to be maintained from upstream exons through to ROSAβgeo* (Schnütgen et al., 2005) (Fig. 1A,B). These modified gene trap cassettes included heterotypic *Frt/F3* and *loxP/lox5171* recombinase target sequences flanking a strong Adenovirus exon 2 splice acceptor site, the enhanced *βge*o* reporter fusion gene and a poly-A termination signal (Friedel and Soriano, 2010, 1991; Schnütgen et al., 2005, 2008). The reading-frame-specific gene trap cassettes were then converted to the following GATEWAY Entry clones: prsFlipROSAβgeo*TT0-ENTR11, prsFlipROSAβgeo*TT1-ENTR11 and prsFlipROSAβgeo*TT2-ENTR11 (Schnütgen et al., 2005, 2008). The traditional gene targeting plasmid, pPNT (Tybulewicz et al., 1991), was modified for use as a GATEWAY Destination Clone (pPNT-DEST-R4-R3). The *Pgk-neo* cassette in pPNT was replaced with the *attR4-ccdB-Cm^r^-attR3* cassette excised from the pDEST-R4-R3 plasmid to allow substitution of the βgeo* gene trap cassette in the final targeted trapping vector. Final assembly of the conditional targeted trapping vector was accomplished via a multi-fragment GATEWAY recombination Clonase reaction. Even with the combination of multiple large DNA fragments into a single targeting construct, this strategy was highly efficient. Twenty-five of 32 (78.1%) ampicillin-resistant transformants contained the desired recombined conditional gene trap plasmid, as revealed by PCR screening (red and black arrows in Fig. 1B) and restriction mapping (data not shown).

The targeted trapping vector prsFlipROSAβgeo*TT0, designed to insert into the second intron of *Prdm16*, was linearized and electroporated into murine J1 129/Sv ES cells and stable transformants were selected in G418 (125 μg/ml). Correct homologous recombinants were identified by long-range genomic- and reverse transcriptase (RT)-PCR (Fig. 1C). Clone 16, producing the expected genomic and mRNA amplification products, was converted into mice by blastocyst injection.

### Germline transmission and complementation testing

Clone 16 was injected into C57BL/6J blastocysts and transplanted into a host mother. Presence of chimeric mice in subsequent litters was evident by mixed coat color and demonstrated contribution by the targeted ES cells. Male chimera were outcrossed to FVB/NJ female mice for germline transmission and for obtaining the mutation in the genetic background on which we maintained the original ENU-induced *Prdm16* mutation. For complementation testing, the chimeric males were bred to heterozygous females carrying the previously described ENU-induced cleft secondary palate 1 (*csp1*) mutation to ensure that the two mutations were allelic (Bjork et al., 2010). The *Prdm16^csp1^* allele carries an intronic point mutation that impairs splicing efficiency to exon 7, resulting in a frameshift and premature termination of the *Prdm16* transcript. We examined newborn pups for the presence of CP to indicate the failure of the conditional gene trap (cGT) *Prdm16^cGT^* allele to complement the *csp1* mutation. Two male chimera mice were bred to *Prdm16^csp1^* heterozygous females, yielding a total of six newborn litters. Four of these litters produced a total of nine pups carrying both the *cGT* and *csp1* allele, and each exhibited CP. This confirms that the cGT cassette in the second *Prdm16* intron is equivalent to a null mutation.

### Validation of the conditional *Prdm16^cGT^* mutation

**Fig. 2.**
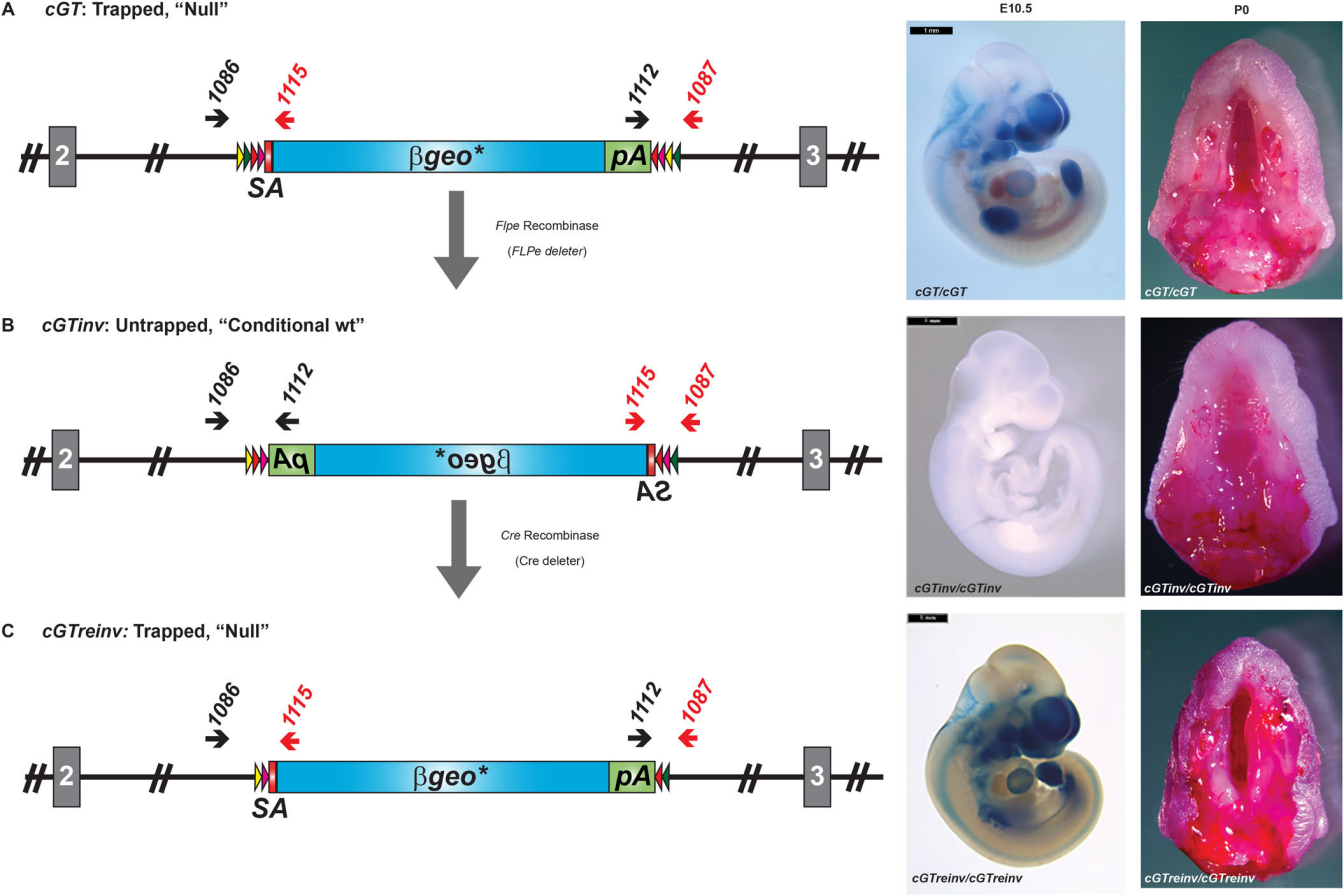
***In vivo* functional validation of the *Prdm16^cGT^* multipurpose allele.** The conditional gene trap (cGT) cassette orientation and recombination endpoint within *Prdm16* intron 2 is depicted on the left with the X-gal staining pattern and palate phenotype shown to the right for each cGT allelic variant (A-C). Note that the same X-gal-stained images used for comparisons in Fig. 3 are included here to illustrate the general βgal expression pattern observed for each cGT allelic variant. (A) Schematic of the *cGT* allele produced by targeted trapping in ES cells (left). Strong X-gal staining in craniofacial tissues, forebrain, hindbrain, dorsal root ganglia, limb and heart is shown in E10.5 embryos to the right along with the cleft secondary palate (CP) evident in the inferior view of the maxilla in P0 homozygous mutant heads with mandibles removed (right). (B) Schematic of the outcome from crossing the *cGT* allele to *FLPe* deleter mice to induce ubiquitous and germline Flp-mediated site-specific recombination (SSR) between like *Frt* or *f3* Flp recombinase target sites to produce the inverted, untrapped conditional allele (referred to as *cGTinv*). Intermediate recombination events in which deletion of one *Frt* and one *f3* site occurs are not depicted (Schnütgen et al., 2005). This allows the inversion to be stabilized even with continued or renewed Flpe exposure. To the right, absence of X-gal staining and CP in *cGTinv* homozygotes confirms the return of endogenous *Prdm16* expression and trap inactivation. This strain is used in combination with Cre-expressing mouse strains for conditional *Prdm16* ablation studies. (C) By crossing mice that carry the *cGTinv* allele to Cre-expressing *BAct*-cre deleter mice, re-inversion of the gene trap cassette occurred between *loxP* or *lox5171* Cre-recombinase target sites. This reconstituted the ‘trapped’ orientation of the cGT cassette (referred to as *cGTreinv*) and left a single *loxP* and *lox5171* flanking the gene trap cassette, which stabilized this inversion. Strong X-gal staining is again observed in craniofacial tissues, forebrain, hindbrain, dorsal root ganglia, limb and heart as shown in E10.5 embryos, and the CP phenotype is evident in P0 homozygous mutants. Wild-type (wt) and *cGT* alleles are genotyped using oligonucleotide primers in the genomic sequence immediately flanking the cGT cassette and primers at the 5′ end of the βgeo* fusion gene or within the *βGH* 3′ polyadenylation (pA) signal (Fig. S1). SA, splice acceptor. Scale bars: 1 mm.

For proof of concept regarding the conditionality of the cGT cassette, we bred heterozygous males (*Prdm16^cGT/+^*) to *Flpe* or *Cre* deleter mice to induce gene trap inversions. Conditionality of prsFlipROSAβgeo* is provided by pairs of heterotypic *Frt/F3* and *loxP/lox5171* recombinase target sites flanking the βgeo* gene trap cassette (Schnütgen et al., 2005, 2008). The original *Prdm16^cGT^* allele produced the CP mutant phenotype in homozygous newborn pups and showed a β-galactosidase (βgal) expression pattern at embryonic day (E)10.5 similar to endogenous *Prdm16* in developing craniofacial primordia, forebrain, hindbrain, heart, limbs and dorsal root ganglia (Fig. 2A). We also confirmed that no *Prdm16* transcripts including exons downstream of the gene trap insertion existed in *Prdm16^cGT/cGT^* mutants, as demonstrated by RT-PCR amplification from total RNA isolated from wild-type (wt), heterozygous and homozygous mutant embryos (Fig. S1). Next, we crossed *Prdm16^cGT/+^* males to homozygous *Flpe* [*Gt(ROSA)26Sor^tm1(FLP1)Dym^*] deleter mice maintained on an FVB/NJ strain background (Farley et al., 2000). As anticipated, double heterozygous mice for the *Prdm16^cGT^* and *Flpe* deleter alleles exhibited gene trap cassette inversion. We will refer to the Flpe inverted *cGT* allele as *cGTinv* (*Prdm16^cGTinv^*). *Prdm16^cGTinv^* heterozygous mice were subsequently bred to FVB/NJ mice to segregate the *cGTinv* and *Flpe* alleles. Heterozygous *Prdm16^cGTinv/+^* mice were bred to homozygosity and maintained by homozygous matings. As expected, cGT inversion restored *Prdm16* and abrogated βgeo* expression, and, most importantly, *Prdm16^cGTinv/cGTinv^* homozygous pups exhibited no abnormal phenotypes (Fig. 2B). To reinduce the original gene trap mutation, we crossed homozygous *Prdm16^cGTinv/cGTinv^* to *βAct-Cre* [*FVB/N-Tg(ACTB-cre)2Mrt/J*] deleter mice (Lewandoski et al., 1997). Double heterozygous mice for the *Prdm16^cGTinv^* and *Cre* alleles exhibited gene trap cassette inversion. We will refer to the Cre inverted *cGTinv* allele as *cGTreinv* (*Prdm16^cGTreinv^*). *Prdm16^cGTreinv/+^* heterozygous mice were subsequently bred to FVB/NJ mice to segregate the *cGTreinv* and *Cre* alleles and were maintained in an FVB/NJ background by successive outcrosses. Heterozygous *Prdm16^cGTreinv/+^* mice were intercrossed to produce homozygous *Prdm16^cGTreinv/cGTreinv^* embryos or pups with reinverted cGT cassettes. Fig. 2C shows that *Prdm16^cGTreinv/cGTreinv^* newborn pups developed CP and re-expressed βgeo*, indicating that the original gene trap mutation could be readily reinduced by Cre-mediated cassette inversion. Overall, we obtained three strain variants carrying the original cGT, the Flpe inverted *cGTinv* and the Cre reinverted *cGTreinv* alleles, which demonstrated that the cGT cassettes are invertible from mutating to non-mutating configurations and back by the consecutive *in vivo* application of Flpe and Cre recombinases.

### Gene trap reporter expression recapitulates endogenous *Prdm16* expression

**Fig. 3.**
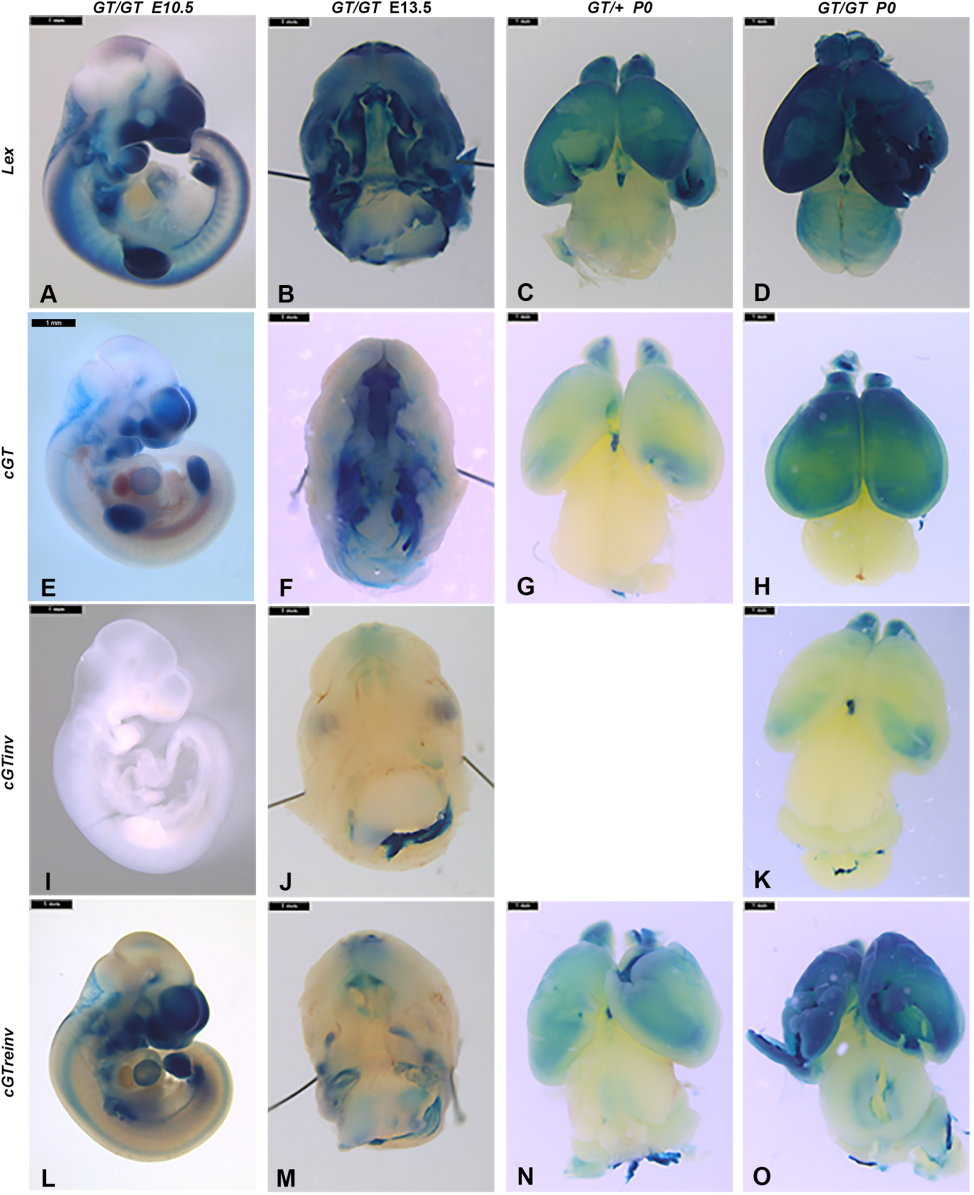
Comparison of βgeo* gene trap reporter expression in *Prdm16^Gt683Lex^* and *Prdm16 cGT*, *cGTinv* and *cGTreinv* alleles. X-gal-stained *Prdm16^Gt683Lex^* (*Lex*) embryos and tissues are shown in the first row (A-D), followed by *Prdm16^cGT^* (*cGT*) (E-H), *Prdm16^cGTinv^* (*cGTinv*) (I-K) and *Prdm16^cGTreinv^* (*cGTreinv*) (L-O). Homozygous E10.5 and E13.5 embryos are compared in columns 1 and 2, followed by heterozygous and homozygous P0 brains. All embryos and whole brains were stained following consistent X-gal staining conditions. Comparison of E10.5 homozygous embryos showed consistency in X-gal staining pattern and intensity in craniofacial tissues, forebrain, hindbrain, dorsal root ganglia, limb and heart between *Lex*, *cGT* and *cGTreinv* embryos (A,E,L) with the expected absence of reporter expression in homozygous *cGTinv* embryos (I). Inferior views of E13.5 embryonic heads with mandibles removed exhibit gene trap allele-specific reporter expression pattern differences. *Lex* homozygotes (B) show strong staining in nasal cartilage, primary and secondary palate, incisor teeth mesenchyme, inner ear and hindbrain choroid plexus (not shown), whereas *cGT* and *cGTreinv* heads (F,M) showed staining in the nasal cartilage, primary palate, incisor teeth mesenchyme, inner ear and fourth ventricle choroid plexus with reduced intensity and, surprisingly, no obvious staining in the secondary palate shelves is evident. In *cGTinv* homozygotes (J), some unexplained background X-gal staining is observed, along with strong staining in the fourth ventricle choroid plexus. Note that the images in E, I and L are also shown in Fig. 2 to represent X-gal staining patterns observed in the three *Prdm16* cGT allelic variants. Scale bars: 1 mm.

To further validate the *Prdm16* gene trap alleles, we performed X-gal stainings on whole-mount E10.5 and E13.5 *Prdm16^Gt683Lex^* [positive control, harboring a conventional gene trap integration in intron 1 of *Prdm16* (Zambrowicz et al., 2003, 1998)], *Prdm16^cGT^*, *Prdm16^cGTinv^* and *Prdm16^cGTreinv^* embryos as well as on postnatal day (P)0 brains. We compared the βgal expression pattern of homozygous *Prdm16^Gt683Lex^* embryos to the patterns exhibited by the *cGT* allelic variants. *Prdm16^Gt683Lex^* E10.5 embryos showed strong staining in the developing forebrain, craniofacial prominences, heart, limbs, hindbrain and dorsal root ganglia (Fig. 3A). Consistent with mutating and non-mutating gene trap configurations, the βgal expression pattern of *Prdm16^cGT/cGT^* and *Prdm16^cGTreinv/cGTreinv^* embryos was comparable to *Prdm16^Gt683Lex^* embryos, whereas *Prdm16^cGTinv/cGTinv^* embryos showed no βgal expression (Fig. 3E,I,L). At E13.5 βgal expression in non-craniofacial tissues of *Prdm16^Gt683Lex^* and *Prdm16^cGT/cGT^* embryos reflected endogenous *Prdm16* expression (Bjork et al., 2010) (data not shown). When the inferior maxilla and secondary palate region were examined in more detail, *Gt683Lex* homozygotes showed strong staining in the nasal cartilage upper incisor and molar mesenchyme, secondary palate and inner ear (Fig. 3B). *Prdm16^cGT^* or *Prdm16^cGTreinv^* embryos exhibited a similar βgal expression pattern except for in the secondary palate, where βgal was not detectable by X-gal staining (Fig. 3F,M). *Prdm16^cGTinv^* embryos showed only weak background X-gal staining and unexplained staining in the fourth ventricle choroid plexus (Fig. 3J). When heterozygous and homozygous pups from each strain were compared, we observed overlapping βgal expression patterns between *Prdm16^Gt683Lex^* and *Prdm16^cGT^* or *Prdm16^cGTreinv^* brains, albeit that βgal expression in *Prdm16^cGT^* or *Prdm16^cGTreinv^* appeared generally weaker. Heterozygous P0 brains (Fig. 3G,N) exhibited weak X-gal staining, whereas X-gal staining of homozygous brains was comparable to *Prdm16^Gt683Lex^* heterozygotes (Fig. 3H,O,C). Strong staining throughout the forebrain and olfactory bulbs was also observed. However, we detected some low-level and variable βgal expression in *Prdm16^cGTinv/cGTinv^* embryos despite the inverted gene trap, most likely due to staining artifacts (Fig. 3K) and not to incomplete inversions, because this allelic variant has been maintained in independent lines for many generations.

**Fig. 4.**
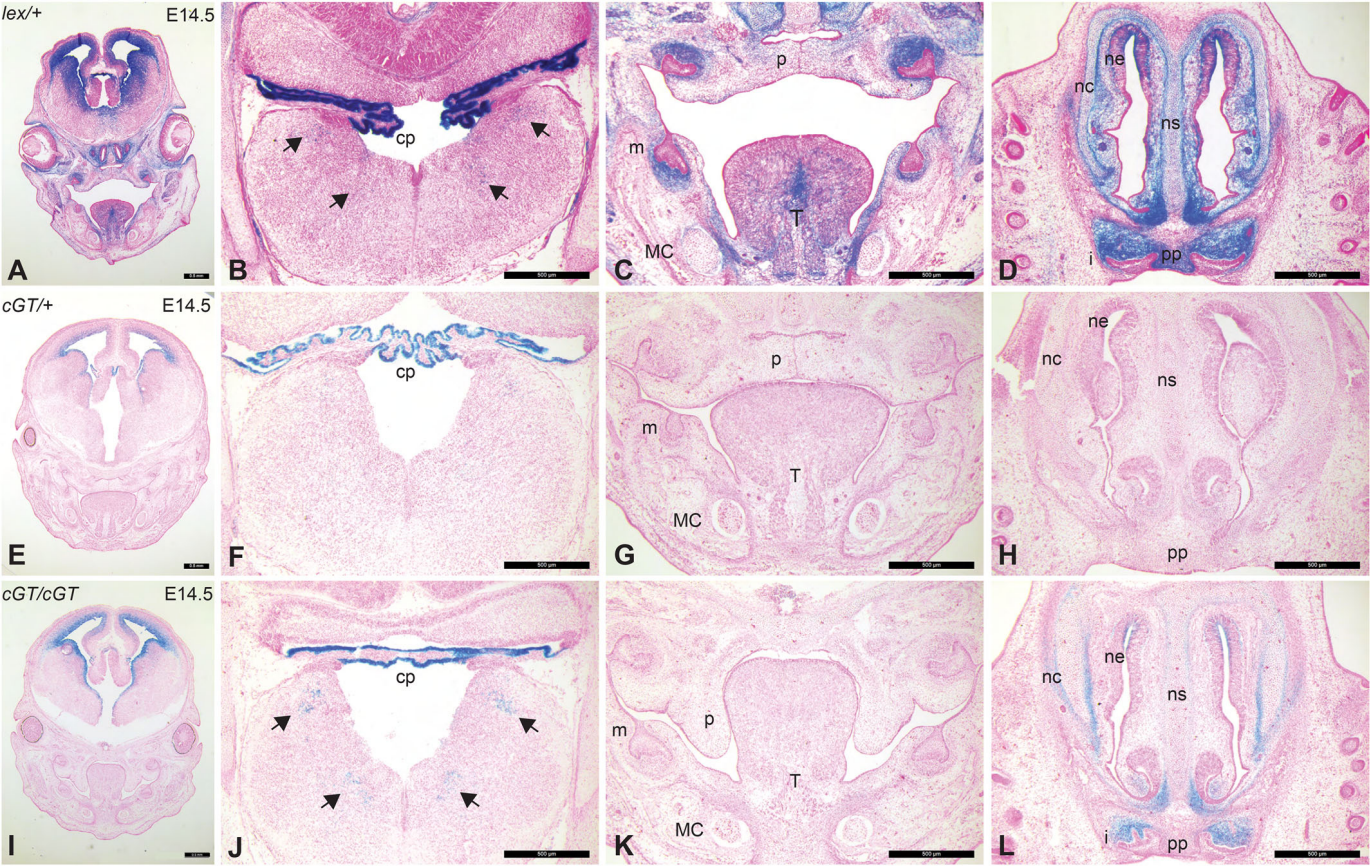
Comparison of βgeo* reporter expression in E14.5 *Prdm16^Gt683Lex^* and *Prdm16^cGT^* embryonic heads. X-gal-stained *Prdm16^Gt683Lex/+^* (*lex/+*) heterozygous head sections are shown in the first row (A-D), followed by *Prdm16^cGT/+^* (cGT/+) heterozygotes (E-H) and *Prdm16^cGT/cGT^* (*cGT/cGT*) (I-L) homozygotes. There is a dramatic reduction in the levels of βgal expression (blue) evident between *lex/+* and *cGT/+* embryos. X-gal staining in *lex/+* heterozygotes is much more intense than in homozygous *cGT* mid-palate head sections (A,I) and in all other tissues examined. It is again notable that the staining pattern is consistent between these *Prdm16* gene trap alleles; however, the expression levels are globally reduced. The regions of highest intensity staining in *lex/+* sections show strong expression in *cGT* homozygotes [i.e. forebrain cortex (A,I), fourth ventricle choroid plexus (cp) (B,J), molar and incisor tooth mesenchyme, and nasal cartilage (nc) and epithelium (ne) (C,D and K,L)]; however, regions of weaker staining show absence of βgal activity in *cGT* homozygotes (i.e. secondary palate shelves in A,C and I,K). Arrows in B and J depict nerve ganglia in the medulla oblongata. Additional abbreviations: Meckel's cartilage (MC), palate shelves (p), primary palate (pp), molar teeth (m), incisor teeth (i), tongue (T) and nasal septum (ns). Scale bars: 500 µm.

To characterize the gene trap expression pattern further, we performed X-gal staining on sectioned *Prdm16^Gt683Lex^* and *Prdm16^cGT^* embryos at E14.5 (Fig. 4). We again compared *Prdm16^Gt683Lex/+^* heterozygous embryos to both heterozygous and homozygous *Prdm16^cGT^* embryos. In *Prdm16^Gt683Lex/+^* heterozygotes, strong X-gal staining was observed in the forebrain cortex, hippocampus and choroid plexus as well as in the eye, nasal epithelium, palate, molar mesenchyme, tongue and Meckel's cartilage (MC). In heterozygous *Prdm16^cGT/+^* embryos, minimal X-gal staining was observed, whereas, in homozygotes, only those structures showing the strongest staining in *Prdm16^Gt683Lex/+^* stained positive for βgal, including the fourth ventricle choroid plexus, ganglia of the medulla oblongata (Fig. 4B,F,J), the molar tooth mesenchyme, the palate shelves, the tongue, the MC (Fig. 4C,G,K), the nasal capsule and the mesenchyme of the upper incisor teeth (Fig. 4D,H,L). Despite strong X-gal staining in the palate shelves of *Prdm16^Gt683Lex/+^*, we did not observe similar staining in *Prdm16^cGT/cGT^* embryos, most likely due to different gene trap vector configurations (Zambrowicz et al., 2003, 1998).

### Phenotypic characterization of *Prdm16^cGT^* mutants

**Fig. 5.**
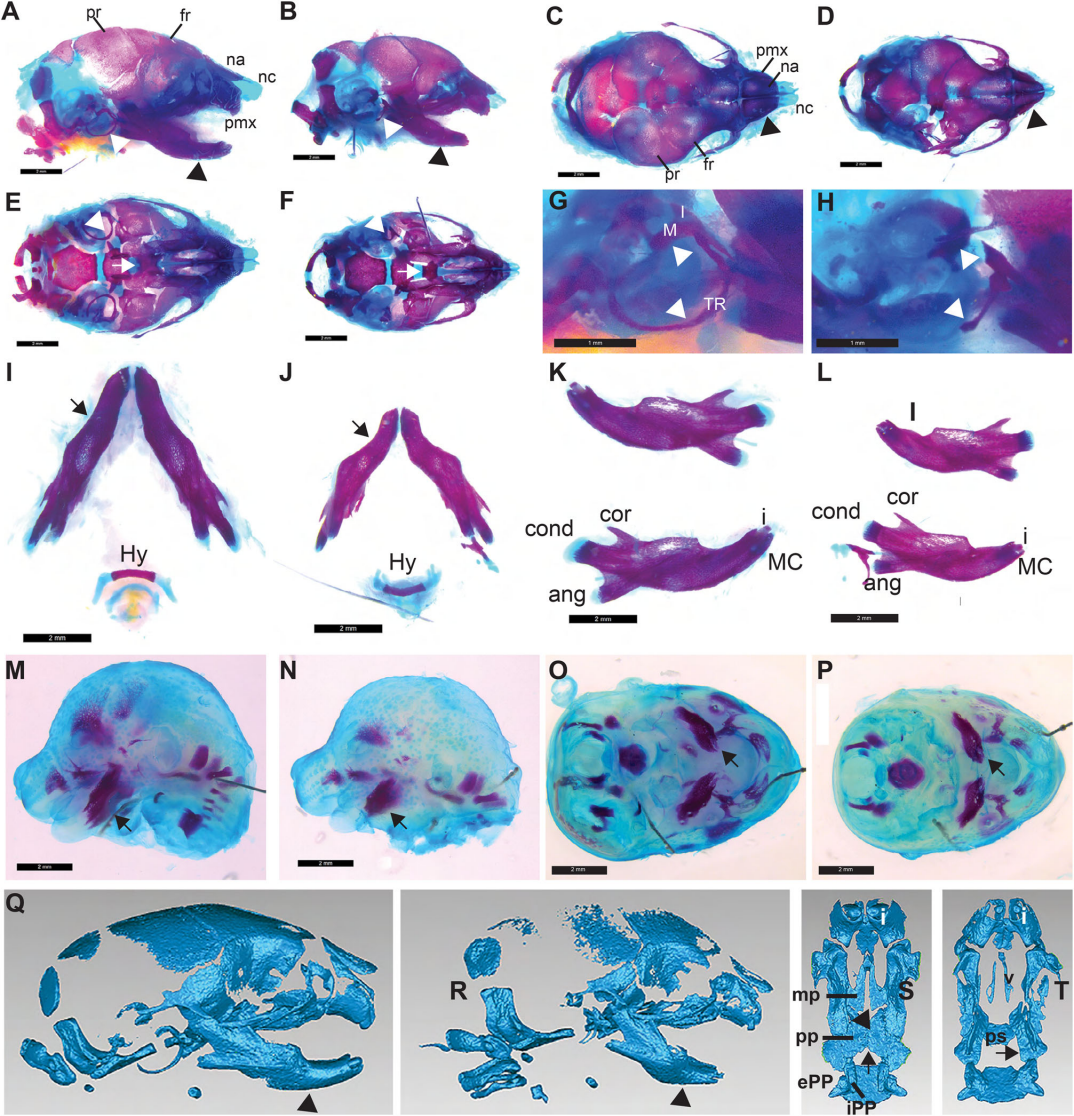
***Prdm16^cGT^* mutants exhibit defects affecting the craniofacial skeleton.** (A-H) P0 skeletal preparations of an unaffected heterozygous (A,C,E) and mutant (B,D,F) head from the same litter stained with Alizarin Red and Alcian Blue; lateral (A,B), dorsal (C,D) and inferior (E,F) views. Abnormalities in the craniofacial skeleton are evident in a general reduction in size of the skull, hypoplasia of the nasal capsule and nasal cartilage (black arrowhead in C,D), mandible (black arrowheads in A,B), tympanic rings (white arrowheads in A,B and E,F) and failed fusion of the palatine bone (white arrows in E,F). (G,H) Magnified view of the lateral tympanic rings illustrate the dramatic hypoplasia and thickening of the superior aspects of the rings in heterozygotes versus mutants (white arrowheads). Malleus and incus exhibit delayed ossification. (I-L) Dissected mandibles and hyoid bones from the same P0 pups. Obvious hypoplasia of the ventral mandible, especially in the anterior aspects where an abnormal curvature is observed, is indicated by the arrows in I and J. No defects in the hyoid bone are evident. This micrognathic phenotype is evident in lateral views of the bisected mandible bone as well (K,L). This anterior shortening accompanies the observation that incisor teeth are reduced in size in mutants as well (K,L). (M-P) Skeletal preparations of E15.5 wild-type (wt; M,O) and mutant (N,P) embryonic heads. Hypoplasia of the mandible is again evident in both lateral (M,N) and ventral (O,P) views and depicted by black arrows. Reduced ossification of the frontal and parietal bones is also observed. (Q-T) Lateral µCT images of wt (Q,S) and mutant (R,T) P0 heads illustrate the micrognathia (black arrowheads in Q,R), hypoplastic tympanic rings and nasal capsule, and decreased ossification seen in homozygous mutants (R) compared to wt controls (Q). The nasal capsule is dysmorphic and the nasal and pre-maxillary bones are hypoplastic and show reduced ossification as well. Inferior view of the maxilla illustrates the failed fusion of the palatine processes that demonstrates cleft secondary palate (CP) in mutants (black arrows in S,T), which makes the vomer (v) and presphenoid (ps) bones visible in mutants. Parietal bone (pr), frontal bone (fr), nasal bone (na), nasal cartilage (nc), pre-maxilla (pmx), hyoid bone (Hy), incisor tooth (i), coronoid (cor), condylar (cond) and angular (ang) processes of the mandible, external pterygoid process (ePP) and internal pterygoid process (iPP), palatine process (pp) and maxillary process (mp), malleus (M), incus (I), Meckel's cartilage (MC), tympanic ring (TR). Scale bars: 2 mm, except for G,H, which are 1 mm.

The phenotype of *Prdm16^cGT^* and *Prdm16^cGTreinv^* mice was virtually identical with the previously reported *Prdm16^Gt683Lex^* and *Prdm16^csp1^* phenotypes (Bjork et al., 2010). Homozygous mutants exhibited fully penetrant wide CP due to failed palate shelf elevation and died shortly after birth due to respiratory failure and abdominal distention. Heterozygous *Prdm16^cGT^* and *Prdm16^cGTreinv/+^* mice showed a low incidence of CP. We extensively characterized the craniofacial phenotype of the *Prdm16* gene trap mutants by histology, skeletal preparations and micro-computed tomography (µCT) scanning. To date, these analyses failed to identify phenotypic differences between mice that carry the *Prdm16^cGT^* or *Prdm16^cGTreinv^* alleles (data not shown); therefore, we used them interchangeably for the characterization of the *Prdm16* null phenotype. Overt CP was evident in P0 pups (Fig. 2A,C), whereas failed palate shelf elevation and tongue flattening was observed much earlier in coronal sections through *Prdm16^cGT/cGT^* heads (Fig. 4G,K). Moreover, MCs were smaller and rounder in *Prdm16^cGT/cGT^* mutants (Fig. 4K) compared with *Prdm16*^*lex/+*^ and *Prdm16*^*cGT/+*^ heterozygotes (Fig. 4C,G). To examine craniofacial bone and cartilage morphology, we stained P0 and E15.5 *Prdm16^cGTreinv/cGTreinv^* mutants and wt controls with Alcian Blue for cartilage and Alizarin Red for bone (Fig. 5). Mutant heads were reduced in size at P0 and micrognathia was observed by gross examination and µCT scanning as well as in skeletal preparations (Fig. 5A-F,Q,R). Moreover, µCT scans showed reduced ossification of the frontal and parietal bones, although this was less obvious in skeletal preparations and nasal cartilage appeared shortened, together with the nasal and pre-maxilla bones (Fig. 5A-D,Q,R). Consistent with the phenotype of the other *Prdm16* mutants (Bjork et al., 2010), *Prdm16^cGTreinv/cGTreinv^* mandibles were hypoplastic, and showed dramatic anterior shortening with abnormal curvature and defective incisor tooth formation (Fig. 5I-L) (Bjork et al., 2010). As shown in Fig. 5M-P, this micrognathic phenotype was already apparent at E15.5. Like in the other *Prdm16* mutants, ossification of the malleus and incus inner ear bones was delayed and tympanic rings were hypoplastic (Fig. 5A,B,Q,E,F,R). A ventral view of the skull with the mandible removed uncovered failed palate fusion (Fig. 5E,F,S,T). Interestingly, loss-of-function Goosecoid (*Gsc*) mutations exhibit a similar phenotype, suggesting that *Prdm16* and *Gsc* are part of a common genetic pathway (Rivera-Perez et al., 1995; Yamada et al., 1995). *Gsc* and *Prdm16* act during TGFβ signaling as well (Chen et al., 1997; Labbé et al., 1998).

### Conditional *Prdm16* ablation in the brain

**Fig. 6.**
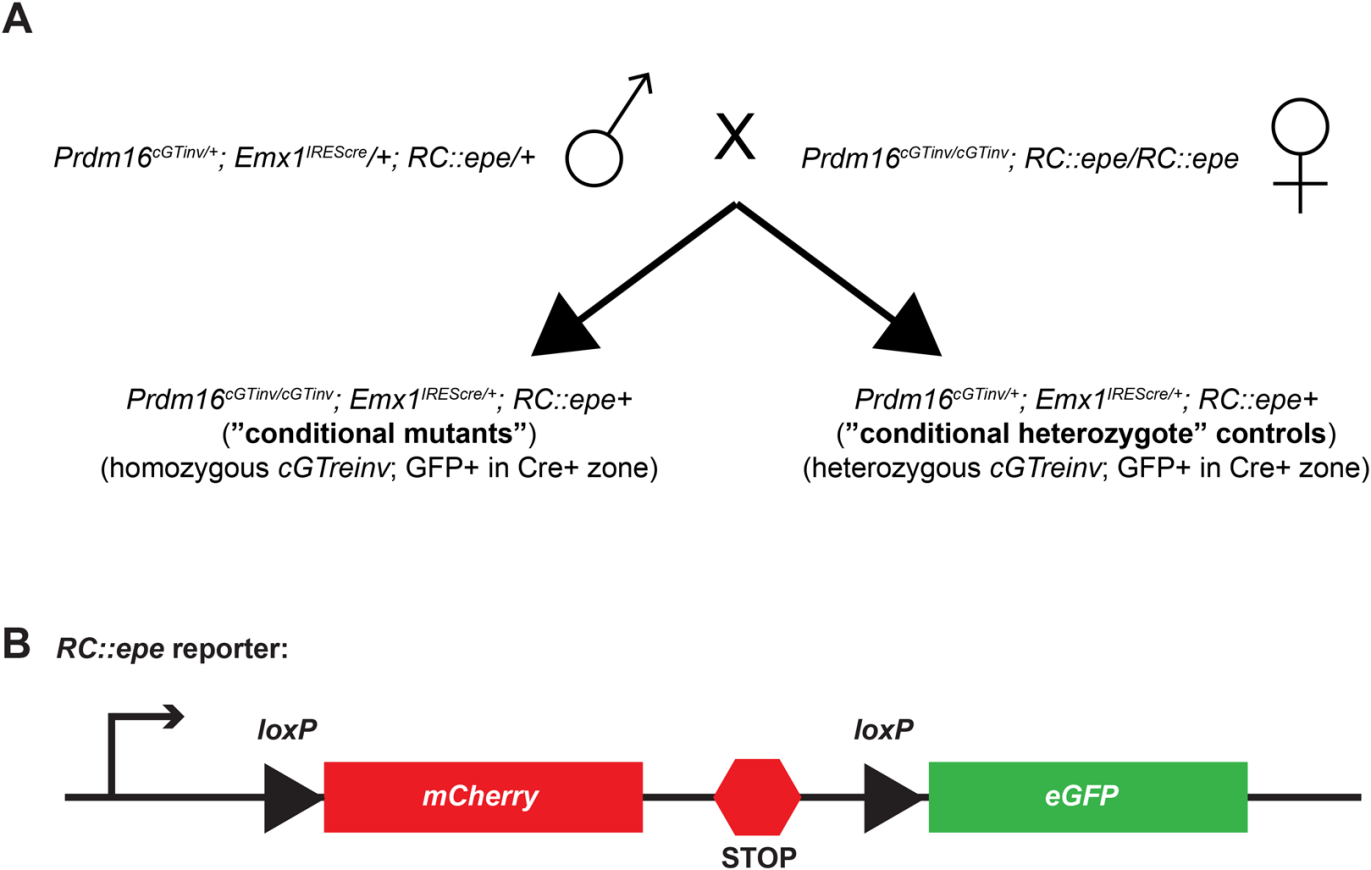
**Conditional breeding scheme and schematic of *RC::epe* Cre reporter allele.** (A) The conditional cross was performed as shown. A homozygous *Emx1^IREScre^* male was bred to *Prdm16^cGTinv^;* *RC::epe* double homozygous females to produce *Prdm16^cGTinv/+^;* *RC::epe/+;* Cre+ mice. These triple heterozygous males were bred to *Prdm16^cGTinv^;* *RC::epe* double homozygous females. Forebrain-specific *Prdm16* ‘conditional mutants’ of the *Prdm16^cGTinv/cGTinv^;* *RC::epe+;* Cre+ genotype and Cre-positive ‘conditional heterozygote’ littermates of the *Prdm16^cGTinv/+^;* *RC::epe+;* Cre+ genotype were used in this study. Cre-mediated inversion of the conditional *Prdm16^cGTinv^* allele to the null *Prdm16^cGTreinv^* allele occurs only in cells in the *Emx1^IREScre^* expression domain. (B) The *RC::epe* Cre reporter allele ubiquitously expresses mCherry in the absence of Cre expression and GFP in the presence of Cre with mutual exclusivity.

*Prdm16* null mutants die shortly after birth, which precludes the phenotypic evaluation of *Prdm16* loss postnatally. We observe a qualitative decrease in forebrain size in *Prdm16^cGT^* null mutant newborn pups compared to controls (data not shown), which is consistent with previous studies performed using the *Prdm16^Gt683Lex^* gene trap null allele (Chuikov et al., 2010). Furthermore, the robust expression of *Prdm16* in the subventricular zone (SVZ) of the forebrain cortex during embryogenesis has been demonstrated (Bjork et al., 2010; Chuikov et al., 2010). To test the utility of the *Prdm16^cGTinv^* allele for conditional ablation studies, as well as to explore *Prdm16* function in the brain and evaluate these conditional mutants as a model for adult neurological disease, we conditionally removed *Prdm16* in the embryonic forebrain by crossing *Prdm16^cGTinv/cGTinv^* mice to the *Emx1^IREScre^* (*Emx1::cre*) deleter strain (Gorski et al., 2002). To analyze this mutation in the background of a Cre activity reporter strain, *Prdm16^cGTinv/cGTinv^* mice were first outcrossed to *Gt(ROSA)26Sor^tm6Dym^* (*RC::epe*) mice expressing a lox-mCherry-lox-GFP reporter transgene from the *Rosa26* locus (Purcell et al., 2009). Carriers of both alleles were bred to homozygosity to obtain *Prdm16^cGTinv/cG^^Tinv^;* *RC::epe* double homozygous offspring (Fig. 6). The *RC::epe* allele directs expression of mCherry in any cell not exposed to Cre recombinase. By excising mCherry from the transgene reporter, Cre induces GFP expression accurately reflecting the sites of Cre expression (Fig. 6B).

**Fig. 7.**
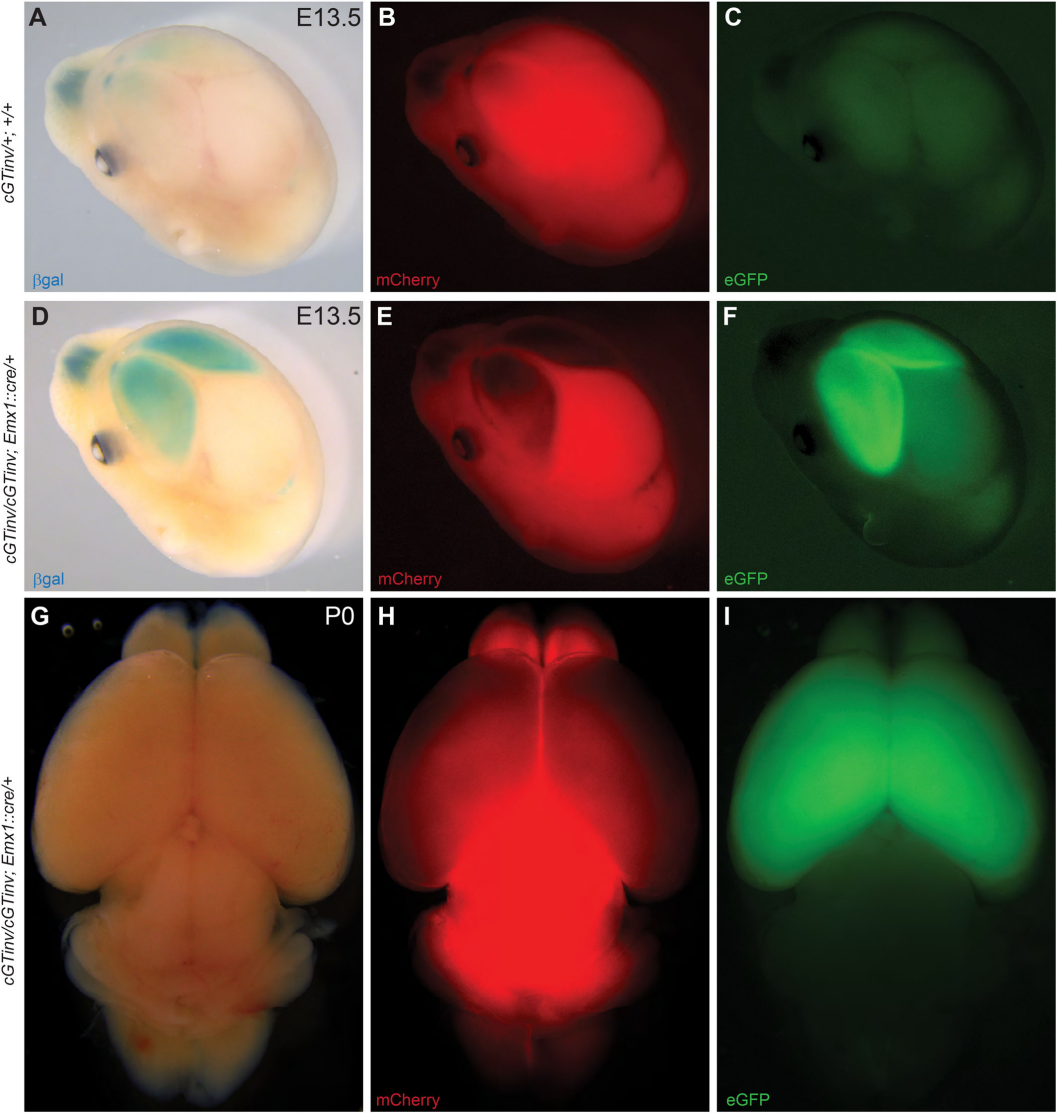
***Emx1^IREScre^* efficiently mediates forebrain-specific inversion of the conditional *Prdm16^cGTinv^* allele.** Validation of forebrain-specific Cre-mediated recombination of the *Prdm16^cGTinv^* allele. Doubly homozygous female conditional *Prdm16^cGTinv^; RC::epe* mice were crossed to *Prdm16^cGTreinv^*^/+^*; Emx1::cre/+* males to generate forebrain-specific conditional *Prdm16* null mutants. (A-F) An X-gal-stained E13.5 *Prdm16^cGTinv/+^; +/+* control head is shown compared to a *Prdm16^cGTinv/cGTinv^; Emx1::cre/+* homozygote showing forebrain-specific *βgal* expression (A,D). Minimal low-level background staining is present in *Prdm16^cGTinv/+^; +/+* control embryos. Ubiquitous mCherry expression and absence of GFP expression in Cre-negative controls is evident (B,C), whereas mCherry expression is reduced in the forebrain where GFP expression is activated in the conditional mutants (E,F). The reduced mCherry expression is obscured by the X-gal staining, since images were taken from the same embryo; however, in a P0 conditional mutant brain that did not undergo X-gal staining, mCherry fluorescence is reduced in the forebrain where GFP is present (G-I).

As shown in Fig. 7A-C, Cre-negative E13.5 heterozygous *Prdm16^cGTinv^^/+^;* *RC::epe+; +/+* controls expressed only minimal amounts of βgal in the nasal region but ubiquitously expressed mCherry, reflecting absent Cre expression. In contrast, Cre-positive homozygous *Prdm16^cGTinv/cGTi^^nv^;* *RC::epe+; Emx1::cre/+* embryos developed strong βgal expression in the forebrain, which overlapped with GFP expression (Fig. 7D-F), indicating that gene trap inversion was restricted to the sites of Cre expression. Fig. 7H,I show that this expression persisted in P0 mutant embryos.

**Fig. 8.**
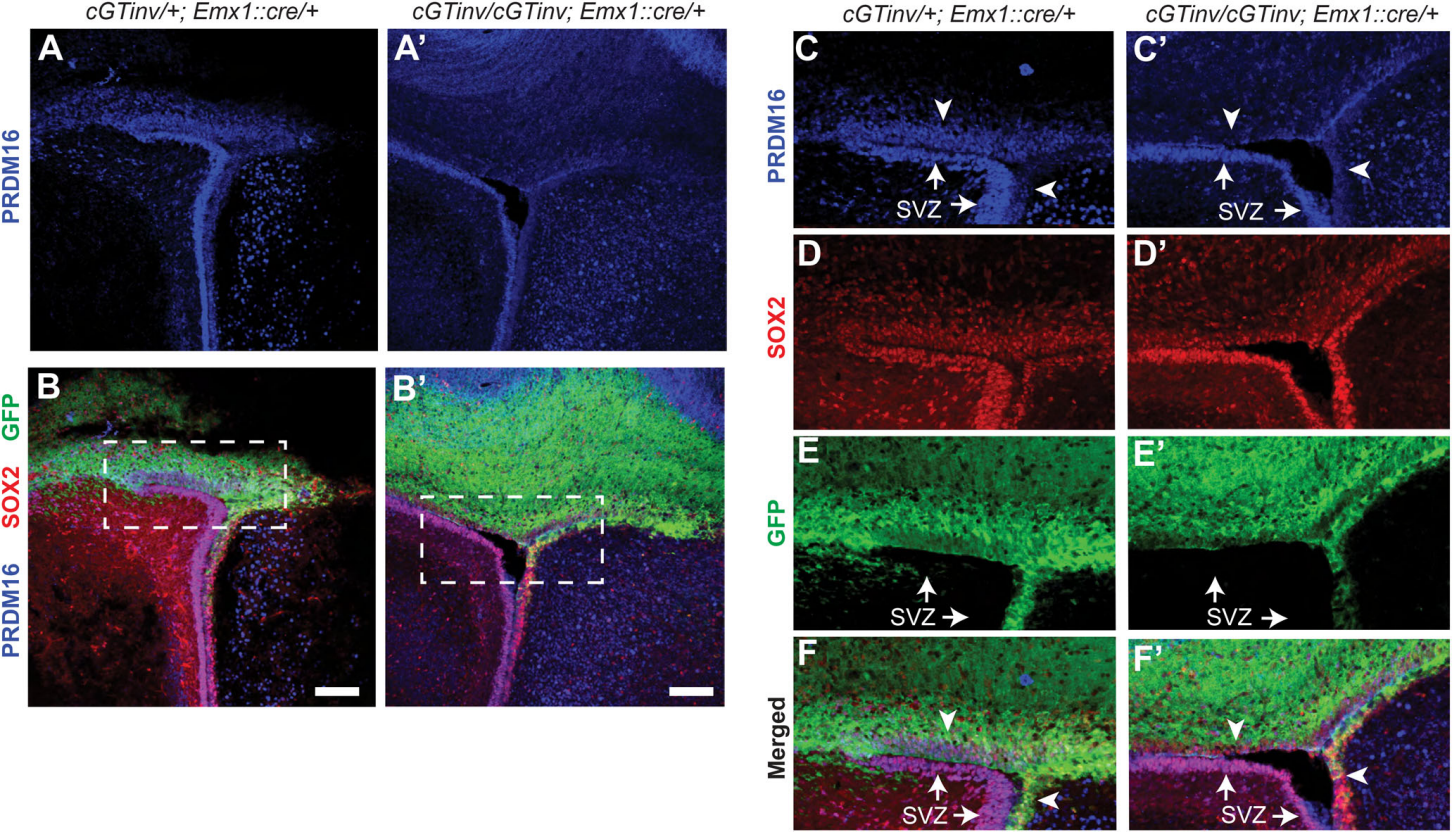
**Validation of the conditional *Prdm16^cGTinv^* allele by forebrain-specific *Prdm16* ablation.** Conditional mating scheme is shown (top). Images of cortices from a P0 conditional heterozygous control pup (*Prdm16^cG^^Tinv/+^;* *RC::epe+; Emx1::cre/+*) (A-F) or from a P0 forebrain-specific conditional mutant pup (*Prdm16^cGTinv/cGTi^^nv^;* *RC::epe+; Emx1::cre/+*) (A′-F′). PRDM16 expression overlaps with SOX2 in the cells of all three lateral ventricle surfaces in the control (A-F). PRDM16 is depleted in the GFP-positive cells in conditional mutants (A′-F′). Note, PRDM16 is still expressed in the GFP-negative ventral surface of the left ventricle and in the sub-ventricular zone (SVZ), demonstrating the efficient conditional ablation of *Prdm16* (white arrows in C,E,F and C′,E′,F′). Dashed rectangles in B and B′ are shown at higher magnification in C-F and C′-F′. Arrowheads indicate the dorsal and medial surfaces of the left ventricle. Scale bars: 50 µm.

PRDM16 expression overlaps with that of the neural progenitor marker SOX2 (Hutton and Pevny, 2011) in multiple neural progenitor cell zones of the E13.5 wt brain (Fig. S2), including the ventricular zone (VZ) and SVZ, the ganglionic eminence (GE) and the hippocampus (HI). Additionally, PRDM16 is strongly expressed in the choroid plexus (CP), where SOX2 is absent. To test whether the spatially restricted gene trap inversions also abolish endogenous *Prdm16* expression, we visualized PRDM16 along with SOX2 in P0 brain sections by immunostaining. Fig. 8A-F shows overlapping PRMD16 and SOX2 expression in all three surfaces of the lateral ventricle (LV) in brains of heterozygous *Prdm16^cGTin^^v/+^;* *RC::epe+; Emx1::cre/+* controls. However, PRDM16 was depleted from the dorsal and medial surfaces of the LV of homozygous *Prdm16^cGTinv/cGTin^^v^;* *RC::epe+; Emx1::cre/+* brains, which closely correlated with the GFP expression pattern (Fig. 8A′-F′,E). Taken together, these results validate the targeted trapping approach as a promising and highly adaptable tool for conditional mutagenesis in mice.

We also examined cell proliferation and apoptosis in our forebrain-specific mutant brains in light of the previous study in *Prdm16^Gt683Lex^* mutant newborns that found reduced cell proliferation and increased apoptosis in the forebrain (Chuikov et al., 2010). We used anti-Ki67 and anti-Caspase-3 antibodies to examine cell proliferation or apoptosis, respectively, and observed no obvious differences in our experiments (data not shown). Interestingly, *Prdm16^cGTinv/cGTin^^v^;* *RC::epe+; Emx1::cre/+* conditional mutants survived to adulthood without obvious gross morphological defects in the brain. However, preliminary histological assessment of the forebrain in these mutants identified heterotopias, possibly resulting from impaired neuronal migration (data not shown). It should be noted that *Prdm16* is only depleted from select regions of the developing forebrain, and its expression in other areas of the brain remains unaffected. By contrast, *Prdm16^Gt683Lex^* mutants represent a complete null condition and therefore exhibit more profound defects. Given the broad expression of PRDM16 in multiple brain regions, it is reasonable to speculate that cell non-autonomous effects from loss of *Prdm16* at other brain regions may contribute to impaired  neural stem cell self-renewal and increased apoptosis observed in the VZ of *Prdm16^Gt683Lex^* mutants.

## DISCUSSION

The present study described a conditional mutagenesis approach enabling mammalian gene functional studies in a spatially and temporally restricted manner. The approach takes advantage of a gene trap amenable to directional inversions by Flpe and Cre recombinases at the insertion sites (Schnütgen et al., 2005). Introduced into a gene of interest by targeted trapping in ES cells that are subsequently converted into mice, the gene trap can be switched from mutagenic to non-mutagenic configurations by breeding to *Flpe* or *Cre* deleter mice. Although the potential of this gene trap for inducing conditional mutations has been known for some time, the present experiments show for the first time that its targeted insertion enables conditional ablation of a pre-specified gene. By using a multi-fragment GATEWAY recombination strategy, we generated targeted trapping constructs for the *Prdm16* gene in as little as 2 weeks. Mice derived from the targeted ES cells readily recapitulated the phenotypes described previously for ENU (*Prdm16^csp1^*) and gene trap (*Prdm16^Gt683Lex^*) *Prdm16* mutations (Bjork et al., 2010).

Homozygous FlipROSA*βgeo* gene trap mutants exhibited CP and micrognathia, reminiscent of the PRS-type cleft palate, as well as gross morphological defects affecting the eye, lungs, heart and brain (data not shown). Conditional ablation of *Prdm16* in the brain of homozygous *Prdm16^cGTinv/cGTinv^* newborn pups that underwent *Emx1^IREScre^-*mediated gene trap re-inversion revealed PRDM16 loss in the forebrain that could be accurately mapped to the dorsal and medial surfaces of the lateral ventricle by the simultaneous visualization βgeo* (X-Gal staining) and Cre (GFP) expression. By using *Prdm16^cGTinv/cGTinv^* mice on a background carrying the *RC::epe* GFP Cre-reporter allele, we could clearly associate loss of PRDM16 in the forebrain with Cre expression and gene trap re-inversion with βgeo* reporter gene expression (Fig. 7). As the forebrain-specific *Prdm16* mutants are viable and in light of the heterotopic forebrain phenotype, they could be used for the analysis of late-onset behavioral and motor coordination phenotypes along with further assessment of associated morphological changes and molecular mechanisms. Heterotopias are commonly observed in various neurological conditions, including epilepsy (Andrade, 2009; Fry et al., 2013). Moreover, by crossing to other tissue-specific Cre-drivers, *Prdm16* gene trap mice can be used to dissect the function of *Prdm16* in brown adipose differentiation, hematopoiesis or neurogenesis, just to name a few of the pleiotropic consequences of *Prdm16* null mutations (Aguilo et al., 2011; Chuikov et al., 2010; Kajimura et al., 2008; Seale et al., 2008, 2007). From a developmental standpoint, the conditional *Prdm16* mutants will be useful to dissect the anatomic and molecular mechanisms underlying the craniofacial abnormalities.

There have been extensive efforts by the IKMC to mutate every single protein-coding gene in ES cells by homologous recombination (Skarnes et al., 2011). Launched in 2003, the project has created a repository of mutant ES cells, most of them conditionals, for nearly 18,000 genes. However, while enabling spatially and temporally controlled knockouts, the IKMC targeted alleles lack a reporter. This complicates mutation mapping to specific cells and tissues by requiring target-protein-specific antibodies, of which many are unsuitable for immunohistochemistry. By providing a reporter, FlipROSA*βgeo* alleles circumvent this need, as the mutations can be directly mapped by simple X-gal staining. Since reporters are exchangeable, replacing *βgeo* with fluorescent reporters, such as those used in this study, may simplify mutation mapping even further. Overall, we strongly believe direct visualization of mutation sites as shown here for the *Prdm16* gene trap mutants embodies a considerable advantage for the characterization of conditional mutations in the mouse.

Although the gene trap cassettes were introduced into the genome by out-of-date technology, it is easily conceivable to use the CRISPR/Cas9 system instead, and in particular the recently reported CRISPR/Cas9 generic gene targeting strategy (Lackner et al., 2015). The latter circumvents homologous recombination and could be used directly for making mutant mice (Wang et al., 2013; Yang et al., 2013). Thus, the combination of targeted trapping with CRISPR/Cas9 generic targeting is likely to provide a one-step approach for the swift production of conditional mouse mutant strains.

## MATERIALS AND METHODS

### Construction of 5′ and 3′ homology arm and gene trap Entry plasmids

5′ (∼5.8 kb) and 3′ (∼2.8 kb) genomic homology regions from the second intron of *Prdm16* were PCR-amplified from MICER clones containing mouse 129/Sv strain genomic DNA inserts using *Pfx* DNA polymerase (Invitrogen - Life Technologies) (Adams et al., 2004). These primers [oBB 1040/1042 and oBB 1043/1044 (5′ and 3′ homology arms, respectively)] contained specific GATEWAY *att* sites [*attB4* and *attB1* (5′ arm) and *attB2* and *attB3* (3′ arm)] at their 5′ ends to allow the amplified homology arms to be recombined into pDONR vectors (pDONR-P4-P1R and pDONR-P2R-P3) via *in vitro* BP Clonase reactions to generate pENTR-5′Hom and pENTR-3′Hom Entry clones (see Table S1). *attL1-attL2*-flanked gene trap entry clones specific to each reading frame [Targeted trap (TT)+0, TT+1 and TT+2], prsFlipROSAβgeo*-TTN-ENTR11 was constructed as follows: pENTR11 plasmid DNA was sequentially digested with *Xmn*I (blunt) followed by *Not*I and treated with Calf Intestinal Phosphatase (CIP). prsFlipROSAβgeo*-TTN (N represents the three reading frame variants) plasmids were digested first with *Apa*I followed by treatment with Klenow fragment to blunt the ends and digestion with *Not*I. Ligation of gel-purified vector and insert created the series of prsFlipROSAβgeo*-TTN-ENTR11 Entry plasmids to allow targeted trapping within any gene intron. All Entry plasmids were transformed into chemically competent *Stbl3* cells (Invitrogen - Life Technologies) and grown on Luria broth (LB) agar plates supplemented with kanamycin.

### Construction of gene targeting GATEWAY Destination vector

We converted the traditional gene targeting vector, pPNT (Tybulewicz et al., 1991) into the pPNT-DEST-R4-R3 GATEWAY Destination plasmid by replacing the *Neo^r^* cassette with a PCR-amplified *attR4-ccdB-Cm^r^-attR3* cassette of pDEST-R4-R3 vector. In brief, pPNT was digested with *Not*I and *Xba*I and CIP-treated followed by gel purification. The *attR4-ccdB-Cm^r^-attR3* cassette was amplified using *Pfx* DNA polymerase and oligonucleotide primers oBB 1054 and oBB 1055 containing 5′ *PspOMI* and *Nhe*I sites, respectively. The product was PCR-purified (Qiagen), digested with *PspOMI* and *Nhe*I and ligated to the digested gel-purified pPNT vector backbone to create pPNT-DEST-R4-R3. Ligations were transformed into chemically competent DB3.1 *E. coli* cells (Invitrogen - Life Technologies) and grown on LB agar plates supplemented with kanamycin.

### Construction of conditional gene trap targeted trapping vector

The three Entry clones, pENTR-5′Hom, pENTR-3′Hom and prsFlipROSAβgeo* TT0-ENTR11, and the pPNT-DEST-R4-R3 Destination vector were combined to create the final targeted trapping vector via the *in vitro* Multisite GATEWAY recombination system using LR Recombinase Plus enzyme following the manufacturer's protocols (Invitrogen - Life Technologies). Plasmid DNA was isolated using the QIAprep Spin Miniprep Kit (Qiagen). The final ∼19 kb targeted trapping vector, prsFlipROSAβgeo*-*Prdm16*in2, was transformed into chemically competent *Stbl3 E. coli* cells and grown on LB agar plates supplemented with ampicillin. Primers spanning 3 of 4 recombination junction sites were used to screen transformants (pUC_F/oBB 1067, oBB 1069/965 and oBB 1073/hsvtk_R; Fig. S1 and Table S2), and positive clones were verified using restriction mapping.

### Conditional gene trap insertion into mouse ES cells

The prsFlipROSAβgeo*-*Prdm16*in2 targeted trapping vector was linearized by *Asc*I digestion and electroporated into J1 129/Sv ES cells. Neomycin-resistant ES cell colonies were grown in G418 (125 μg/ml or 150 μg/ml) for 10 days. G418-resistant colonies were picked and split into replicate plates for: (1) frozen storage in 10% DMSO/DMEM; (2) X-gal staining; (3) genomic DNA isolation; and (4) total RNA isolation. X-gal staining was performed in culture wells following protocols described elsewhere in this manuscript. Genomic DNA was isolated using standard Proteinase K digestion protocols (Nagy et al., 2003). Total RNA was isolated using Trizol Reagent following the manufacturer's protocols (Invitrogen - Life Technologies). The promotorless gene trap depends upon endogenous *Prdm16* expression in ES cells to confer G418 resistance, so titrations of G418 concentrations were tested to determine optimal levels. For our experiment, 125 μg/ml G418 was sufficient to allow correctly targeted ES cells to survive, while still killing most incorrectly targeted ES cells.

To identify correctly targeted ES cell colonies, total RNA was extracted using Trizol Reagent and used as template in One-Step RT-PCR assays using primers oBB 1116 and oBB 1125 to amplify a predicted 360 bp product and identify the desired *Prdm16*-βgeo* fusion gene trap transcript (Fig. 1C,D; Invitrogen - Life Technologies). Of the 80 ES cell lines resistant to 125 μg/μl G418, two were PCR-positive. Genomic DNA extracted from RT-PCR-positive ES clones were screened for desired recombination events using primers (oBB 1112/1113) designed near the 3′ end of the gene trap cassette and outside the 3′ homology arm sequence and *Pfu* Ultra II Fusion HS DNA Polymerase (Stratagene) to generate a 3.3 kb PCR product in correctly targeted clones (Fig. 1C,D). Correct 5′ targeting was verified only in ES clones that were positive by RT-PCR and the 3′ targeting event. These primers (oBB 1114/1115) were designed near the 5′ end of the gene trap cassette and outside the 5′ genomic homology arm sequence and yielded a 6.1 kb product (Fig. 1C,D). Correctly targeted ES cells containing the cGT cassette insertion into *Prdm16* intron 2 were microinjected into C57BL6/J blastocysts (Brigham & Women's Hospital, Partners Healthcare Transgenic Core facility, Boston, MA). Germline chimeras were obtained and crossed to *Prdm16^csp1/+^* mice (Bjork et al., 2010) for complementation testing or FVB/NJ mice to monitor germline transmission. Germline transmission was confirmed by failure to complement the *csp1* mutation, agouti coat color in heterozygous progeny from the FVB/NJ outcross and confirmation by PCR genotyping to identify the *Prdm16^cGT^* allele. This *Prdm16^cGT^* allele is available on request. All animals were housed in accordance with Harvard Medical School (HMS) or Midwestern University (MWU) Animal facility regulations, and all studies were performed consistent with protocols approved by the HMS or MWU Institutional Animal Care and Use Committees.

### Genomic DNA isolation and genotyping

Genomic DNA was isolated from individual embryos and weaned mice using a modified ‘HotSHOT’ protocol (Truett et al., 2000). A total of 2 µl of the ∼500 µl supernatant was used as template for allele-specific PCR genotyping assays. Allele-specific genotyping assays were designed to differentiate the *cGT* alleles. Oligonucleotide primers outside the cGT cassette (proximal, oBB 1086; distal, oBB 1087) were paired with primers located at the 5′ end of the βgeo* fusion gene (oBB 1115) or within the bovine growth hormone (*bGH*) 3′ polyadenylation signal (oBB 1112). Use of one flanking genomic primer paired with the two internal primers will yield a product of a specific size dependent upon the orientation of the gene trap cassette at the locus (Fig. S1). Primers specific for the wt *Prdm16* allele (oBB 1086/1087), trapped *Prdm16^cGT^* and *Prdm16^cGTreinv^* gene trap alleles (oBB 1087/1112) or untrapped conditional *Prdm16^cGTinv^* allele (oBB1087/1115) were used (Fig. 2 and Table S2). Amplified products were electrophoresed through 2% agarose gel and documented using a ChemiDoc Imaging System (Bio-Rad Inc.). oBB 1086/1087 amplifies the wt allele only and yields a 370 bp product. oBB 1087/1112 is specific for the trapped orientation of the *cGT* cassette and yields the following products in the various allelic variations due to deletion of *loxP* or *Frt* sites during intermediate site-specific recombination events: *cGT*=724 bp, *cGTinv*=NA, *cGTreinv*=508 bp. Similarly, oBB 1087/1115 amplifies only the inverted conditional *cGTinv* allele and yields the following products: *cGT*=NA, *cGTinv*=603 bp, *cGTreinv*=NA. The products amplified become smaller with each inversion event, since *Frt* or *loxP* sites are lost from the cGT cassette by the stable endpoint of Cre- or Flpe-mediated recombination (Schnütgen et al., 2005). Genotype status of embryos or mice derived from forebrain-specific ablation studies was determined by the combination of GFP expression, *cGT* allele genotyping PCR and the presence of the *Cre* allele (oBB 1324/1325; Table S2). In addition, to identify conditional mutants, we screened for absence of the oBB 1086/1087 wt allele, thus demonstrating the presence of two cGT cassettes in these animals. The other allele-specific assays served as amplification controls against these ‘no amplification’ samples being the result of dirty, dilute or degraded DNA.

### Conditionality of the gene trap

Stable inversion and re-inversion of the conditional gene trap occurred as described (Schnütgen et al., 2005). Male heterozygous carriers of the *cGT* allele (*Prdm16^cGT/+^*) were crossed with mice homozygous for the *Flpe* [*FVB/N-Gt(ROSA)26Sor^tm1(FLP1)Dym^*] deleter allele maintained on an FVB/NJ strain background (Farley et al., 2000). Subsequent *Flpe*-positive, *Prdm16^cGT/+^* carriers were outcrossed to FVB/NJ to segregate the *Flpe* allele. *Prdm16^cGT/+^* carriers of the next generation carried the inverted *Prdm16^cGTinv^* conditional allele*.* We bred the *129Sv-Gt(ROSA)26Sor^tm6Dym^* (*RC::epe*) Cre reporter allele (Purcell et al., 2009) into this strain and intercrossed the resulting double heterozygous males and females to double homozygosity (*Prdm16^cGTinv/cGTinv^; RC::epe/RC::epe*) for maintenance of the strain, since the *Prdm16^cGTinv^* allele harbors no obvious abnormal phenotype. These female mice are routinely used in conditional ablation crosses. Finally, *Prdm16^cGTinv/cGTinv^; RC::epe/RC::epe* mice were crossed to *βAct-Cre* [*FVB/N-Tg(ACTB-cre)2Mrt/J*] (Lewandoski et al., 1997) Cre deleter mice to re-invert the gene trap in the *Prdm16^cGTinv^* allele to the *Prdm16^cGTreinv^* trapped orientation. Subsequent *Cre*-positive, *Prdm16^cGTreinv/+^* carriers were outcrossed to FVB/NJ to segregate the *Cre* allele. The *Prdm16^cGT^* and *Prdm16^cGTreinv^* null alleles have been maintained via repeated outcrosses to the FVB/NJ strain and are considered equivalent models of the ubiquitous loss of *Prdm16* (Fig. 2).

129Sv-*Gt(ROSA)26Sor^tm6Dym^* (*RC::epe*) homozygous mice were generously provided by Dr Susan Dymecki (HMS, Boston, MA). Female 6- to 8-week-old *Prdm16^cGTinv/cGTinv^;* *RC::epe/RC::epe* mice were bred to *B6.129S2-Emx1^tm1(cre)Krj/^*^J^ [*Emx1^IREScre^* (Gorski et al., 2002)] homozygous males to produce *Prdm16^cGTreinv^; RC::epe; Emx1^IREScre^* triple heterozygotes. Male mice of this genotype were backcrossed to the *Prdm16^cGTinv/cGTinv^; RC::epe/RC::epe* females to carry out the conditional ablation cross. Forebrain-specific ‘conditional mutants’ (*Prdm16^cGTreinv/cGTrein^^v^;* *RC::epe+; Emx1^IREScre/+^*) and Cre-positive ‘conditional heterozygote’ controls (*Prdm16^cGTreinv/^^+^;* *RC::epe+; Emx1^IREScre/+^*) each resulted in the expected Mendelian ratios (one-fourth for each including genotypes that contained either one or two *RC::epe* alleles) (Fig. 6A).

### Embryo processing for whole-mount and section X-gal staining of gene trap embryos or pups

Embryos and newborn P0 pups were generated through intercross timed matings between carriers of one of the three *Prdm16 cGT* allele variants. Females were inspected for the presence of a post-copulatory vaginal plug each morning: noon on the day the plug was identified was considered E0.5. Embryos were dissected via post-mortem cesarean section, and yolk sac or tail tissue was removed for DNA isolation and genotyping. Embryos to be used for histological analysis were fixed with Bouin's fixative for at least 48 h, rinsed thoroughly in multiple changes of 70% ethanol and processed for paraffin embedding either manually or with the use of a Leica TP1020 automated tissue processor. Sections were cut at a thickness of 7 or 10 µM and stained with hematoxylin and eosin using standard protocols. Whole-mount and section X-gal staining was performed as described (Nagy et al., 2003). E10.5 embryos were fixed in 2% formaldehyde, 0.2% glutaraldehyde for 30 min on ice, E13.5 embryos for 1 h and P0 brains for 1.5-2 h. For X-gal staining of sections, samples were infused with 30% sucrose for at least 24 h, followed by cryoembedding in OCT and cutting into 15 µm sections for staining.

### Skeletal preparations

Newborn wt and *Prdm16^cGT^* mutant embryos were collected, skinned and eviscerated before placement into 95% ethanol for at least 1 day. All steps were performed with slow rocking at room temperature. Ethanol was replaced with Alcian Blue staining solution (0.03% Alcian Blue, 80% ethanol, 20% acetic acid) for 2-3 days, followed by a 6-12 h wash in 95% ethanol. Ethanol was replaced with 2% KOH solution for 24 h or until the tissue was appropriately cleared, followed by staining in Alizarin Red solution (0.03% Alizarin Red, 1% KOH, water) for 24 h. Skeletons were cleared in 1% KOH/20% glycerol solution and transferred to a 1:1 glycerol:95% ethanol solution. E14.5 embryos were collected and placed immediately into 95% ethanol for at least 1 day. Staining was performed as described above but with reduced time at each step. Images were captured as described previously and using the Leica Montage module within the Leica application suite (LAS) software package.

### Micro-computed tomography (µCT)

Newborn heads were harvested and processed as described for the immunolabeling experiments in the brain. The mice crania were scanned using a Skyscan 1272 µCT system housed at the University of Iowa College of Dentistry. To provide sufficient resolution and image quality, we used a 360° scan protocol at 70 kv and 142 mA with an 18-μm voxel size (resolution). Scans were acquired using a 0.5 mm aluminum filter with a rotation step of 0.6°. These parameters were selected to optimize scan resolution, image quality and scan time. Images were decimated and visualized using Geomagic Studio 2014 (www.3dsystems.com). Three-dimensional volume-rendered models of each cranium were created from the µCT image stacks [3D Slicer v 4.6 (www.slicer.org)].

### Microscopy and imaging

Gross, whole-mount stained and fluorescent embryos and skeletal preparations were visualized using a Leica M165 C stereodissecting microscope equipped with fluorescent capabilities. Stained embryo sections were visualized using a Leica DM 5500 B upright compound light microscope. Digital images were captured using a Leica DFC 450 C color camera or Leica DFC 365 FX monochrome camera using LAS with the Montage module or LAS X software for fluorescence imaging.

### Brain sample preparation for immunolabeling

Embryos and brains dissected from newborn pups were rinsed three times in PBT (PBS/0.1% Tween-20), followed by fixation by immersion in 4% paraformaldehyde in PBT and rinsing and storage in PBT/05% sodium azide. The fixed tissues were sectioned coronally (80-100 μM) using a vibratome (Leica Microsystems).

### Immunofluorescence and confocal imaging

To stain with various antibodies, brain sections were incubated for 1 h at room temperature in blocking solution (10% FBS, 0.3% Triton X-100), then in the primary antibody at 4°C overnight. Sections were washed 3×20 min in 0.3% Triton X-100 in PBS, then incubated in Alexa-Fluor-conjugated or DyLight-conjugated secondary antibodies for 2 h at room temperature. Incubation with DAPI for 5 min and 3×20 min 0.1% Triton X-100 washes followed, both at room temperature.

Primary antibodies used in this study were: rabbit polyclonal anti-PRDM16 (1:500; Bjork et al., 2010) and goat anti-SOX2 (1:150; R&D Systems), mouse anti-TUJ1 (1:1000; Millipore), rabbit anti-Ki67 (1:500; Abcam) and rabbit anti-Caspase-3 (1:200; Cell Signaling). The secondary antibodies used were: donkey anti-rabbit-647 (1:1000; Jackson ImmunoResearch), donkey anti-goat-549 (1:1000; ImmunoResearch) and donkey anti-mouse-647 (1:1000; Jackson ImmunoResearch). Imaging was done on a Leica CTR6500 confocal laser-scanning microscope. Volocity (ImproVision) and Photoshop (Adobe Systems) softwares were used for image processing.
